# Rapid Detection of Staphylococcal Enterotoxin-B by Lateral Flow Assay

**DOI:** 10.1089/mab.2019.0028

**Published:** 2019-10-10

**Authors:** Robert Hnasko, Alice V. Lin, Jeffery A. McGarvey

**Affiliations:** ^1^ USDA-ARS Produce Safety and Microbiology Research Unit (PSM), Albany, California.; ^2^ USDA-ARS Foodborne Toxin Detection and Prevention Research Unit (FTDP), Albany, California.

**Keywords:** monoclonal antibodies (mAb), *Staphylococcus aureus*, Staphylococcal enterotoxin-B (SEB), lateral flow assay

## Abstract

A cohort of monoclonal antibodies (mAbs) were generated against Staphylococcal enterotoxin-B (SEB) and selected by double sandwich enzyme-linked immunosorbent assay (ELISA) for solution capture of the toxin. Clonal hybridoma cell lines were established and a pair of anti-SEB mAbs selected for the development of a sandwich ELISA. Immobilized 3D6 mAb (IgG1, kappa) when paired with 4C9 mAb (IgG1, kappa) conjugated to horseradish peroxidase generates a typical dose–response curve with an EC_50_ of 24.8 ng/mL for purified SEB using chemiluminescent detection. These mAbs bind SEB by Western blot and ELISA binding to classical enterotoxin serotypes show that the 3D6 mAb binds both SEB and the SEC1 serotypes, whereas 4C9 binds only SEB. These mAbs effectively port onto lateral flow test strips with a visual detection sensitivity for SEB of 5 ng/mL in <10 minutes using a 4C9 conjugated to a 40 nm gold reporter.

## Introduction

*Staphylococcus aureus* is a pathogenic gram-positive bacterium that can produce an impressive collection of protein toxins.^(1–3)^ These secreted toxins represent virulence factors and staphylococcal foodborne poisoning (SFP) is a leading cause of foodborne illness in the United States.^(4–6)^ The gastrointestinal (GI) illness associated with SFP is rarely life threatening and the disease is usually self-resolving without hospitalization.^(7)^ However, the economic cost and lost productivity associated with SFP warrants effective control strategies.^(8)^

The staphylococcal enterotoxins (SE) represent a large group of structurally similar and serologically distinct proteins (22–29 kDa) encoded in prophages, plasmids, and chromosomal pathogenicity islands.^(5,9)^ There are five classical antigenic types (A–E) and these superantigens elicit an immune response that results in the massive production of inflammatory cytokines.^(10–12)^ SEB is considered the most dangerous as it is produced by most *Staphylococcus aureus* strains.^(7,13,14)^ SEB is a primary cause of SFP after ingestion^(15,16)^ and is considered a military incapacitating agent as it is highly toxic, thermally stable, and can cause intoxication by inhalation if aerosolized.^(17,18)^

SEB intoxication is difficult to distinguish from other GI illnesses and there is no vaccine and has limited treatment options.^(13)^ There are many immunoanalytical technologies available for SEB detection, but a need remains for portable, rapid, and inexpensive methodologies to address foodborne contamination.^(19,20)^ Commercially produced lateral flow test strips in general report 5–10 ng/mL detection sensitivities using optical readers^(21,22)^ and their applicability is primarily directed toward emergency first responders. In this article we report the generation of a novel cohort of anti-SEB monoclonal antibodies (mAbs) and identify a suitable pair for the development of a sandwich enzyme-linked immunosorbent assay (ELISA) with application in a lateral flow assay format.

## Materials and Methods

### SEB mAbs

Female Balb/cByJ mice (Jackson Laboratory, ME) were immunized by intramuscular injection of an SEB toxoid derived from purified SEB toxin (Sigma, MO) mixed 1:1 with TiterMax gold adjuvant (Sigma). Hybridomas were generated by chemical fusion with P3X myeloma cells and screened by double sandwich ELISA against purified native SEB (Toxin Technology, FL) using a biotinylated rabbit-a-SEB pAb (Toxin Technology) with an avidin-horseradish peroxidase (HRP) reporter and chemiluminescent detection. Hybridoma cell cloning was performed by limiting dilution and total of 24 hybridoma cell lines producing anti-SEB mAbs were isolated. All animal experiments were performed with institutional approval and followed national guidelines for the care and use of laboratory animals.

### Sandwich ELISA

Anti-SEB mAbs were purified on protein-G and a functional pair of anti-SEB mAbs was identified for the development of a sandwich ELISA. In brief, the capture mAb (3D6; IgG1, kappa) was immobilized at 2 μg/mL on black 96-well high-binding polystyrene plates at 5 μg/mL in 0.1 M carbonate buffer (pH 9.4); washed repeatedly in Tris-buffered saline with 0.1% Tween-20 (TBST; pH 7.2) and blocked in 10% nonfat dry milk (NFDM). The SEB antigen was diluted in TBST containing 0.1% BSA and added to wells for 1 hour. The detection mAb (4C9; IgG1, kappa) conjugated to HRP was added at 1 μg/mL for 1 hour. Chemiluminescent substrate (PicoECL; Pierce) was added and luminescent signal recorded as counts per second using a Victor X^3^ luminometer (PerkinElmer). All reactions were performed at room temperature with a minimum of three replicates. Analysis was performed using four parameter logistic (4PL) with dynamic curve fitting (EC_50_ = 24.8 ng/mL; Hillslope = 0.85).

### Western blotting

The SEB antigen was diluted in sample buffer, heat denatured, and 0.5 μg separated on a 4–12% Bis-Tris Gel and protein transferred to a nitrocellulose membrane. Membranes were washed in TBST, blocked with 10% NFDM, incubated with 1 μg/mL of primary antibody then secondary anti-mouse IgG conjugated to HRP. Antibody binding was resolved by chemiluminescence and Tiff images captured using a FluroChem HD2 (Alpha Innotech, CA). Molecular weight was estimated using prestained dual-color protein standards (BioRad, CA).

### Lateral test strips

In brief, RP membrane (Millipore) was striped using a noncontact BioJet HR value with a high-resolution syringe pump attached to an XYZ3050 platform (BioDot, CA) with the 3D6 capture mAb as a test line (T) and a donkey-anti-mouse IgG used for the control line (C). The RP membranes were water washed, then blocked in polyvinylpyrrolidone (PVP40; Sigma) and dried. The 4C9 mAb was conjugated to 40 nm gold (InnovaCoat Gold; Innova Biosciences) and 10 OD sprayed onto a 10 mm glass fiber conjugate pad (Millipore) using a noncontact AirJet HR aerosol dispenser (BioDot) attached to the XYZ platform. Dried membranes were adhered to 60 mm plastic backing card with 25 mm Fusion-5 membrane (GE Healthcare) as a sample pad and 22 mm CF6 membrane (Millipore) as an absorbent sink. The test strips were cut (60 × 4.5 mm) and housed in a two-part plastic cassette with a pressure point at material overlap. Dilutions of SEB were added to the sample pad (100 μL) and resolved for 10 minutes and then photographed.

## Results and Discussion

We have isolated and cloned 24 anti-SEB producing hybridoma cell lines by double sandwich ELISA. Our screening and selection assay utilized purified SEB in its native conformation emphasizing solution capture capability of the mAbs. Most of these mAb show a high degree of SEB binding selectivity and perform in a variety of immunoassay formats that include sandwich ELISA, direct ELISA, Western blotting and lateral flow. Some of these mAbs evaluated by ELISA against the classic SEs (A–E) show binding to the SEC1 serotype that shares the most amino acid sequence identity (68%) with the SEB protein.^(23)^

**FIG. 1.**
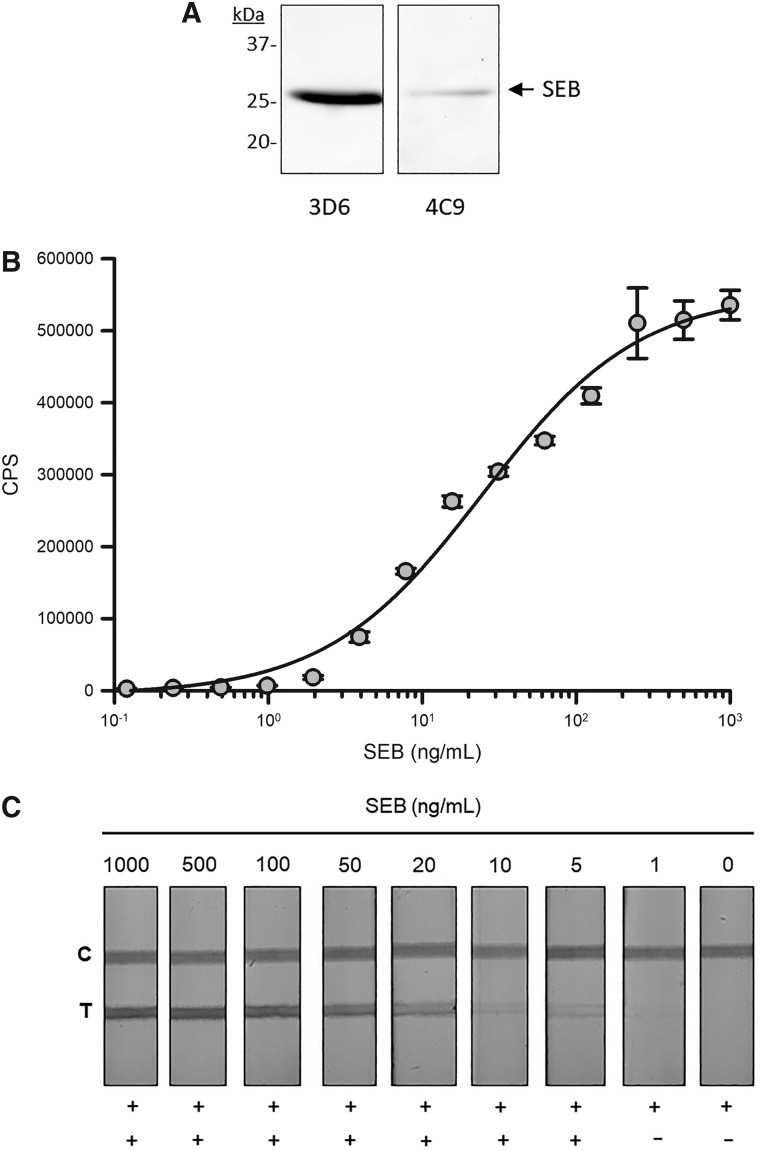
The detection of 28 kDa SEB by Western blot **(A)** using the 3D6 mAb (left panel) and 4C9 mAb (right panel). The dose-dependent detection of SEB by chemiluminescent sandwich ELISA **(B)** using immobilized 3D6 mAb for capture and 4C9-HRP for detection. The line represents 4PL dynamic curve fitting of data with an EC_50_ = 24.8 ng/mL SEB and 0.85 hillslope. Detection of SEB by lateral flow assay **(C)**. SEB is visually detected (+) at the test line (T) using immobilized 3D6 mAb with 4C9 mAb conjugated to 40 nm gold. The test line shows a dose-dependent change in visible test line intensity with 5 ng/mL as the tests limit of detection. A donkey-anti-mouse IgG is immobilized at control line (C) and validates test performance. No test line is observed in the absence of SEB (−). 4PL, four parameter logistic; ELISA, enzyme-linked immunosorbent assay; HRP, horseradish peroxidase; mAb, monoclonal antibody; SEB, Staphylococcal enterotoxin-B.

A pair of anti-SEB mAbs (3D6 and 4C9), with IgG1 heavy chains and kappa light chains, was identified for assay development (Table 1). The 3D6 mAb binds both SEB and SEC1, whereas the 4C9 mAb binds only SEB in ELISA (*data not shown*). These mAbs bind purified heat denatured SEB protein by Western blot (Fig. 1A). To develop the sandwich ELISA the 3D6 mAb was immobilized and used for SEB capture with the 4C9 mAb used for detection. A typical dose–response curve was observed using purified SEB dilutions with an EC_50_ of 24.8 ng/mL of SEB and a hillslope of 0.85 using 4PL dynamic curve fitting (Fig. 1B). These two mAb both function in the sandwich ELISA format as either a SEB capture or detection reagent (*data not shown*).

**Table 1.** The 3D6 and 4C9 Anti-Staphylococcal Enterotoxin-B Monoclonal Antibodies Were Paired for Development of a Sandwich Enzyme-Linked Immunosorbent Assay and Construction of Lateral Flow Test Strips

*Mouse no.*	*mAb*	*Format*	*Isotype*	*Ascites*
223	3D6	Capture	IgG1, kappa	Yes
224	4C9	Detector	IgG1, kappa	Yes

These mAbs were further evaluated by indirect ELISA against the staphylococcal enterotoxins A–E with the 3D6 mAb binding SEB and SEC1 and the 4C9 mAb binding only SEB.

ELISA, enzyme-linked immunosorbent assay; mAb, monoclonal antibody; SEB, Staphylococcal enterotoxin-B.

To develop a rapid SEB detection assay these mAbs were ported on standard 60 × 4.5 mm lateral flow test strips with the 3D6 mAb immobilized at a test line (T) and 40 nm gold-conjugated 4C9 as a SEB reporter. A donkey-anti-mouse IgG was immobilized at the control line (C) and functions to validate the proper performance of the test strip. A dilution series of purified SEB was prepared, 100 μL was applied to the test strip sample pad, and the test allowed to resolve for 10 minutes and then photographed. Visually observable test lines indicating detection of SEB were observed down to 5 ng/mL (Fig. 1C). No test line was observed in the absence of the SEB analyte.

Although the 3D6 mAb will bind the SEC1 serotype, when paired with the selective 4C9 mAb the assay will only detect the SEB serotype. This serotype specificity would address concerns regarding SE cross-reactivity reported with some commercial assays.^(24)^ Many commercially available SEB lateral flow assays fail to report the sensitivity of their tests, whereas other require optical readers to achieve 5–10 ng/mL SEB detection. In this article we report a lateral flow assay that achieves 5 ng/mL SEB detection sensitivity by visual observation. Further optimization of these SEB-specific reagents in a lateral flow assay format along with the integration of an optical reader will likely result in an increase in detection sensitivity and assay performance suitable for commercialization.
